# Unilateral alacrimia as a presenting symptom of Meckel’s cave tumour

**DOI:** 10.3205/oc000241

**Published:** 2024-06-25

**Authors:** Ignacio Manuel López Miñarro, Laura Prieto Domínguez, Víctor Manuel Asensio-Sánchez

**Affiliations:** 1 Hospital Clínico Universitario, Ophthalmology Department, Valladolid, Spain

**Keywords:** middle cranial fossa, trigeminal nerve diseases, alacrimia

## Abstract

Meckel’s cave tumour, a rare benign tumour originating from the Schwann cells surrounding the trigeminal nerve within the Meckel’s cave region, can present with a variety of clinical manifestations. We report a case of a 44-year-old male patient who presented with symptoms of tear deficiency, including dryness, ocular discomfort, and blurred vision. Diagnostic evaluation revealed the presence of a Meckel’s cave tumour harming the trigeminal nerve, leading to alacrimia. This case highlights the association between Meckel’s cave tumour and tear deficiency disorders.

## Introduction

Hypolacrimia or alacrimia is an unusual symptom that requires a thorough evaluation. Hypolacrimia or alacrimia could be due to several mechanisms: firstly, congenital anomalies (agenesia, aplasia, or hypoplasia); secondly, alteration in maturation of the gland; thirdly, tear production relies on the integrity of the proteins involved in the secretory lacrimal system; and lastly, innervation affecting lacrimal gland afferent and/or efferent nerve transmission [1], [2], [3]. The authors report an uncommon presentation of unilateral alacrimia as the presenting symptom of Meckel’s cave tumour without orbital invasion. To our knowledge, this is the first report of alacrimia as the initial symptom of Meckel’s cave tumour.

## Case description

**Figure 1:**
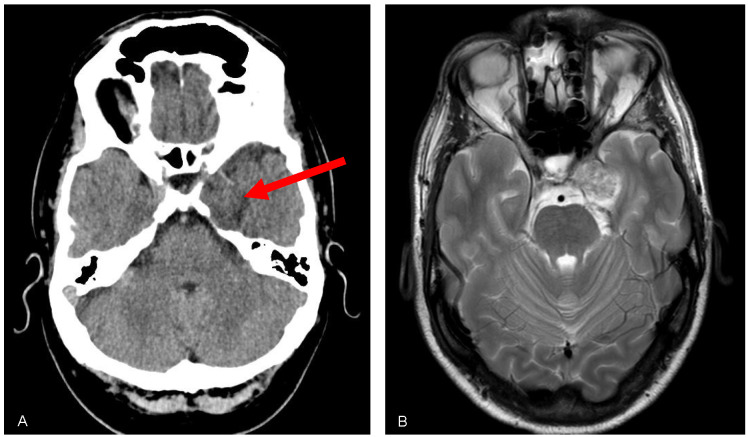
A: Note the lesion on CT scan (red arrow), which remodels the adjacent bone structures. B: MRI shows a nodular T2 enhancing mass in the region corresponding to the trigeminal fossa.

The case involves a 44-year-old male patient who presents with a history of persistent dryness, itching, and redness in the left eye. The symptoms have progressively worsened over the past six months, significantly impacting the patient’s quality of life. On examination, his visual acuity was 20/20 in both eyes (OU). Slit-lamp examination showed superficial punctate keratitis in the left eye, with “practically” absence of tear meniscus, and measurement of the basal tear secretion was 20 mm in the right eye (OD) and 5 mm in the left eye (OS). The rest of his examination was otherwise normal. A computed tomography (CT) of the brain and orbits revealed an extra-axial mass of 3 cm maximum diameter located in the left cavernous sinus, occupying the ipsilateral Meckel’s cave, with heterogeneous density and intensely enhanced after contrast administration (Figure 1A (Fig. 1)). The magnetic resonance imaging (MRI) showed a nodular image of heterogeneous signal occupying and expanding the left Meckel’s cave, predominantly hyperintense in T2 sequences, measuring approximately 27x27x23 mm (transverse x anteroposterior x craniocaudal). It reshapes Meckel’s cave and displaces the internal carotid artery anteriorly, superiorly and to the right, preserving its caliber and patency. It protrudes into the prepontine cistern, where it is in close contact with the V cranial nerve and seems to depend on it. After the administration of contrast, it enhances intensely but heterogeneously, with hypocaptive areas in its interior suggesting schwannoma of the V pair (Figure 1B (Fig. 1)). The patient finally underwent stereotactic radiotherapy, and his left tear production improved. 

## Discussion

**Figure 2:**
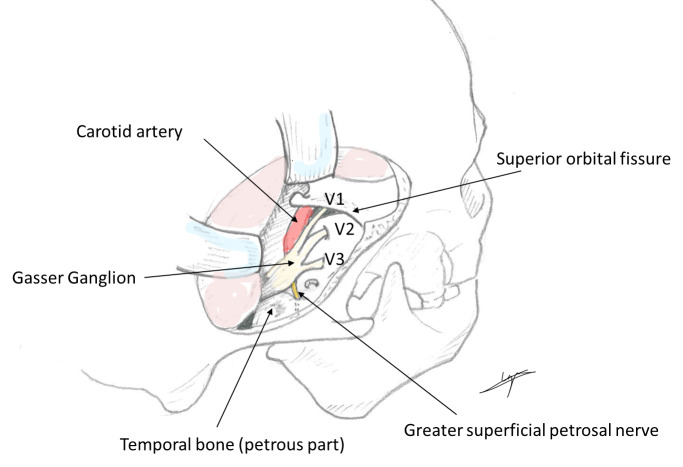
Anatomy of Meckel’s cave. Image of own elaboration, inspired by Sun et al. [6]

Hypolacrimia and alacrimia are rare features in which different mechanisms can be involved. For a minority of conditions the pathophysiology is clearly established but for most of them the existing explanations remain insufficiently proven. Alacrimia is frequently associated with dysautonomia, but also with genetic diseases, such as anhidrotic ectodermal dysplasia, Sjögren’s syndrome, and Allgrove’s syndrome, or may be secondary to trauma or surgery. Acquired alacrimia is an unusual symptom that requires further investigation. Patients with tumours in the trigeminal fossa (or Meckel’s cave), located in middle cranial fossa, may have absence of tear production as a presenting symptom, because through this space it also runs the greater petrosal nerve, inferior to the trigeminal ganglion of Gasser (see Figure 2 (Fig. 2)). This nerve carries parasympathetic fibers for the lacrimal gland, and may be compromised, being in this case the most probable etiology. The greater superficial petrosal nerve, or just greater petrosal nerve, comes from the geniculate ganglion of the 7^th^ cranial nerve, runs posteriorly together with the deep petrosal nerve, and forms the vidian nerve, until it synapses in the pterygopalatine ganglion, from where fibers exit through the zygomatic nerve, and then the zygomatic-temporal nerve, reaching the lacrimal gland together with a division of the ophthalmic branch of the 5^th^ cranial nerve, the lacrimal nerve. The sensory component of the eye might also be affected by an involvement of the efferent fibers from the lacrimal gland. There is a similar case described in which alacrimia is the presenting symptom of a nasopharyngeal carcinoma infiltrating the vidian nerve [1]. Surgical disruption along this pathway, as demonstrated in neurectomies of the vidian nerve, has been shown to eliminate the nasolacrimal reflex and decrease basal tear production [4]. There is another reported case of alacrimia and arhinorrhea in a patient diagnosed with petroclival chondrosarcoma extending to Meckel’s cave and the cavernous sinus [5]. By presenting this clinical case study, we aim to contribute to the existing knowledge on hypolacrimia and alacrimia, shedding light on the diagnostic intricacies and therapeutic challenges encountered in these rare but clinically significant conditions. Improved understanding and recognition of these disorders can lead to timely interventions, ultimately improving patient outcomes and enhancing their quality of life.

## Conclusion

While Meckel’s cave tumours typically present with symptoms related to trigeminal nerve compression, such as facial pain, numbness, or cranial nerve deficits, in some cases, they can also lead to tear deficiency disorders, including hypolacrimia and alacrimia. Understanding the association between Meckel’s cave tumours and tear deficiency is essential for timely diagnosis and appropriate management. 

## Notes

### Competing interests

The authors declare that they have no competing interests.
